# Ehlers-Danlos Syndrome, Hypermobility Type: An Underdiagnosed Hereditary Connective Tissue Disorder with Mucocutaneous, Articular, and Systemic Manifestations

**DOI:** 10.5402/2012/751768

**Published:** 2012-11-22

**Authors:** Marco Castori

**Affiliations:** Division of Medical Genetics, Department of Molecular Medicine, San Camillo-Forlanini Hospital, Sapienza University, Circonvallazione Gianicolense, 87, 00152 Rome, Italy

## Abstract

Ehlers-Danlos syndrome, hypermobility type, constituting a phenotypic continuum with or, perhaps, corresponding to the joint hypermobility syndrome (JHS/EDS-HT), is likely the most common, though the least recognized, heritable connective tissue disorder. Known for decades as a hereditary condition with predominant rheumatologic manifestations, it is now emerging as a multisystemic disorder with widespread manifestations. Nevertheless, the practitioners' awareness of this condition is generally poor and most patients await years or, perhaps, decades before reaching the correct diagnosis. Among the various sites of disease manifestations, skin and mucosae represent a neglected organ where the dermatologist can easily spot diagnostic clues, which consistently integrate joint hypermobility and other orthopedic/neurologic manifestations at physical examination. In this paper, actual knowledge on JHS/EDS-HT is summarized in various sections. Particular attention has been posed on overlooked manifestations, including cutaneous, mucosal, and oropharyngeal features, and early diagnosis techniques, as a major point of interest for the practicing dermatologist. Actual research progresses on JH/EDS-HT envisage an unexpected link between heritable dysfunctions of the connective tissue and a wide range of functional somatic syndromes, most of them commonly diagnosed in the office of various specialists, comprising dermatologists.

## 1. Introduction

**Figure 1:**
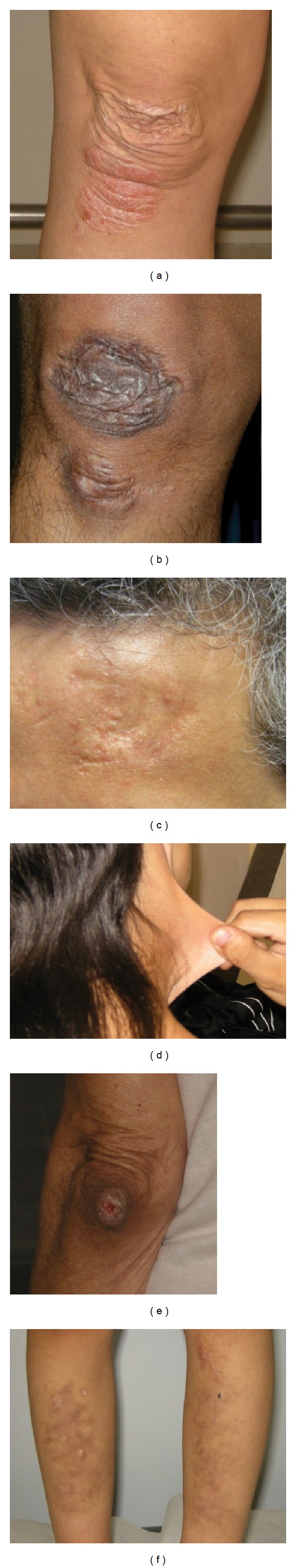
Typical cutaneous features of Ehlers-Danlos syndrome(s). Papyraceous (a), hemosiderotic and atrophic (b), and depressed (c) scars. Skin hyperextensibility (d). Molluscoid pseudotumor of the heel (e). Multiple ecchymoses with hemosiderotic depositions (f). Note that, except for skin hyperextensibility, such cutaneous changes are not observed in the hypermobility type.

Ehlers-Danlos syndrome (EDS) was first recognized in the first decade of the twentieth century as a hereditary disorder with typical skin manifestations (Figure 1) [1, 2]. Over the decades, EDS emerged as a clinically and genetically heterogeneous group of disorders, including an increasing number of variants (Table 1) which share the variable combination of dermal fragility, internal organ and vessel ruptures, and joint hypermobility (JHM) [3]. After many years of nosologic confusion, a group of experts, who met in Villefranche in 1997, identified six major EDS subtypes, namely, classic, hypermobility (i.e., Ehlers-Danlos syndrome, hypermobility type—EDS-HT), vascular, kyphoscoliotic, arthrochalasis, and dermatosparaxis, recognized by specific diagnostic criteria [4]. Among them, EDS-HT is the most difficult to recognize due to the lack of clinical diagnostic handles and confirmatory laboratory/molecular tests. Nevertheless, EDS-HT is now considered the commonest EDS variant [5] with an unexpectedly high disability potential [6].

While EDS-HT is characterized by the “absence” of the typical cutaneous manifestations observed in many other EDS subtypes, skin and mucosae represent common sites of disease manifestation with a plethora of minor anomalies, whose detection still has an invaluable role in suspecting such a creeping condition. Moreover, given the protean constellation of additional features of EDS-HT, mastering the broad spectrum of subtle findings detectable at inspection is crucial for early diagnosis and management of potentially disabling complications. 

## 2. Definition(s)

EDS-HT is a hereditary connective tissue disorder (HCTD) defined by the association of generalized JHM, joint instability complications, widespread musculoskeletal pain, and (minor) skin features [4].  Table 2 summarizes the Villefranche diagnostic criteria for the six best known variants, including EDS-HT. EDS-HT shows a significant phenotypic overlap with the joint hypermobility syndrome (JHS), a rheumatologic disorder with a high disability potential [7] and strong familial aggregation. Whether such similarities reflect etiological identity remains to be established. However, many researchers are used to consider EDS-HT and JHS one and the same (i.e., JHS/EDS-HT) [8], while others do not completely agrees with this assumption [5]. In parallel with EDS-HT, JHS is actually recognized by the revised Brighton criteria (Table 3) [9]. Based on literature data and personal experience, the author of this paper agrees with the concept of considering EDS-HT and JHS the same condition. 

Besides skin manifestations that are relatively unspecific in both sets of diagnostic criteria, (generalized) JHM is the most clinically relevant feature of JHS/EDS-HT. The term “joint hypermobility” refers to the ability of one or more joints to actively and/or passively move beyond normal limits. It may affect a few joints (i.e., monoarticular or localized JHM) or present in multiple body sites (i.e., generalized or general JHM). JHM is a sign, not a disease, and its occurrence varies by age, sex, and ethnicity. In fact, loose joints are more common among females and children than males and older people [10]. By ethnicity, JHM is observed in up to 35–57% Africans [11], while it shows a much lower rate (6% in females, 2% in males) among Caucasians [12]. In addition, various acquired/environmental factors, such as traumas, surgery, and regular training, may contribute in increasing range of motion at one or more joints. 

Interpretation of generalized JHM is not always straightforward and needs a holistic perspective. In fact, JHM is often experienced as an asset for some occupational and sport activities, such as ballet, gymnastics, and playing instruments [13]. At the same time, generalized JHM is the physical marker of various HCTDs. Distinguishing between benign, asymptomatic JHM and an HCTD is of utmost importance for preventing potential life-threatening complications and/or early detecting and managing long-term disabilities. 

## 3. Epidemiology

The early literature fixed to 1/5,000 the frequency of EDS as a whole [14], with EDS-HT accounting for approximately half of all registered cases. However, as JHS/EDS-HT is a neglected HCTD, its frequency is likely underestimated. Accordingly, based on registered data on the frequency of generalized JHM in various populations and the assumption of an ~10% chance of developing symptoms according to the Brighton criteria for “double-jointed” people [12], a presumed frequency of 0.75–2% has been proposed for JHS [15]. As general JHM is rarer among Caucasians compared to other populations such as Africans [16], a frequency of 0.2–0.6%, with the lowest value better fitting for men and the highest for females, appears more realistic in Europe and USA. However, no systematic study accurately investigating the real incidence of JHS/EDS-HT has been performed to date. 

## 4. Etiology

### 4.1. Inheritance Pattern

Actually, JHS/EDS-HT is considered an autosomal dominant trait with complete penetrance [17]. Accordingly, an affected individual may transmit the disease to his/her children with a 50% chance, irrespectively to sex. Nevertheless, such an assumption is not always confirmed by practice. In fact, the Brighton and, perhaps, Villefranche criteria for JHS/EDS-HT are met more commonly by females with a markedly skewed sex ratio [18]. This implies that females are more commonly and, possibly, severely affected than males, and, in familial cases, the disease is more frequently transmitted by an affected mother. In addition, extended family study often shows the coexistence of different members with a clinical diagnosis of JHS, EDS-HT, or asymptomatic JHM (either present or historical), as well as asymptomatic nonhypermobile “carriers” in the same pedigree (Castori, unpublished data). Therefore, JHS/EDS-HT should be better defined as an autosomal dominant trait with incomplete penetrance, variable expressivity, and influenced by sex. On a reproductive perspective, while the “mutated gene” is transmitted from an affected parent to the conceptus with a 50% chance, the likelihood of developing the disease seems to be higher in a female fetus. 

Recently, it has been introduced that in JHS/EDS-HT families asymptomatic JHM could be the core inherited trait, which eventually evolves in JHS/EDS-HT in those members in whom other independent factors converge. Among them, there are both “intrinsic” and environmental/acquired contributors, such as sex, age, somatotype/weight, sport habits, traumas, surgery, diet, and pain cognitions [19]. Therefore, what we actually call “JHS/EDS-HT” could represent the tip of an iceberg with still unclear relationships with physiology (e.g., generalized, asymptomatic JHM) and organ/tissue-specific functional somatic syndromes possibly linked to an underlying connective tissue disorder (see Section 9). 

### 4.2. Molecular Basis of EDSs

Most EDS subtypes are caused by mutations in gene encoding collagen chains or proteins involved in their biogenesis. Biomechanical consequences of an altered collagen fiber in the expressing tissues and differential expression of the various affected collagen subtypes in the tissues are the major determinants for clinical distinction among EDS variants. Most classic EDS (EDS type I and II of the earlier classification) patients harbor heterozygous mutations in the genes encoding for two of the three chains constituting the ubiquitous collagen type V (*COL5A1* and *COL5A2*) [20]. Conversely, >95% of the cases of vascular EDS are due to mutations in the gene encoding collagen type III (*COL3A1*) [21], which is markedly expressed in vessels. Four EDS variants (i.e., arthrochalasia, classic with vascular rupture, cardiac-valvular, and EDS/osteogenesis imperfecta overlap) are caused by dominant or recessive mutations in genes encoding the two chains of collagen type I (*COL1A1* and *COL1A2*) [22–25]. 

Some EDS variants are caused by mutations in proteins/enzymes involved in collagen I biogenesis. Dermatosparaxis EDS is due to abnormalities in* ADAMTS2* [26], which encodes for an N-proteinase involved in the ablation of N-propeptides whose cleavage is essential for complete maturation of collagen I. Hydroxylation of lysine residues of collagens I and II depends on lysyl hydroxylase 1, encoded by *PLOD1*, which mutated causes kyphoscoliotic EDS [27]. 

In patients with the rare tenascin X-deficient EDS due to mutations in* TNXB* [28], immunostaining on muscle biopsies shows mildly reduced expression of collagen VI. Studies on tenascin X null mice show that deficiency of this protein decreases mRNA expression of C*OL6A1*, *COL6A2* and* COL6A3 *[29]. Mutations in these genes, in turn, cause the Bethlem myopathy and Ullrich congenital muscular dystrophy, which are inherited muscle disorders sharing some features, such as atrophic scars and JHM, with EDSs [30]. 

Finally, some rare EDS subtypes, including the progeroid and musculocontractural EDSs, are caused by mutations in genes coding for enzymes involved in the biosynthesis of proteoglycans, which are components of the CT extracellular matrix, exhibiting tight relationships with collagen fibers [31, 32]. The same cellular mechanism may be involved in the novel kyphoscoliotic EDS due to recessive mutations in *FKBP14*. In fact, this gene encodes for a FK506-binding peptidyl-prolyl cis-trans isomerase, which may act as a chaperone altering the assembly of the extracellular matrix [33].

### 4.3. Molecular Basis of JHS/EDS-HT

In contrast to the other EDS variants, the genetic defect underlying JHS/EDS-HT remains unknown. In the past, a handful of papers tried to clarify the conundrum. In particular, some molecular investigations suggested that *TNXB* heterozygous or homozygous mutations can be identified in ~5% of the EDS-HT patients [34, 35]. Subsequently, EDS patients harboring mutations in* TNXB* have been classified in a different EDS subtype (i.e., *TNXB*-deficient EDS) due to an apparently distinct phenotype [36]. A single family considered affected by EDS-HT was found with a mutation in the *COL3A1 *gene, which, in turn, is typically mutated in the vascular EDS [37]. No subsequent study confirmed this preliminary finding. Therefore, neither *TNXB* nor *COL3A1* can be at the moment considered “the gene” of JHS/EDS-HT. Future studies are urgently needed in order to clarify this point and shed more light on the nosologic distinction between JHS and EDS-HT [5]. 

## 5. Clinical Manifestations

JHS/EDS-HT differs from other EDS variants due to the apparent paucity and nonspecificity of clinical findings. This reflects only in part the apparently minor involvement of cutaneous and musculoskeletal connective tissue in this EDS subtype. In fact, the scarcity of descriptive manifestations of JHS/EDS-HT in the medical literature lays on the actual lack of shared knowledge and general unawareness of the practitioners on the multifaceted manifestations of JHS/EDS-HT. The conundrum is further complicated by the increasing number of studies highlighting (generalized) JHM as a possible predisposing factor and/or noncasually associated features for a series of extra-articular disorders (Table 4) [38–64]. At the moment, whether these complaints belong to the wider picture of the JHS/EDS-HT or rather represent nonsyndromic associations needs further investigations. 

### 5.1. Cutaneous Features (Figure 2)

Skin hyperextensibility is certainly the best known cutaneous feature of the various EDSs. In JHS/EDS-HT, it may be appreciated in many patients but usually in a much minor extent compared with classic EDS. Skin hyperextensibility defines the ability of the skin to be stretched beyond normal limits and immediately returning to its original state without forming transient redundant folds. Rapid returning to the original state after traction (i.e., resilience) differentiates skin hyperelasticity from cutis laxa, which can equally be noted with an increased rate in older patients with various EDSs, including JHS/EDS-HT. Cutis laxa is indeed a late consequence of premature elastolysis due to reduced dermal resistance to extreme soft-tissue distensions, such as pregnancy and repeated gains and losses of weight. Premature blepharoptosis/chalasia and chubby checks are typical localized manifestations of cutis laxa and may represent relevant aesthetic complaints in middle-aged women. Impaired stiffness of the dermis caused by defective collagen fibrils can also facilitate the development of striae rubrae and striae distensae/atrophicae. Similarly, piezogenic papules (i.e., small spontaneous subcutaneous fat herniations through a defective dermis without appreciable dermal atrophy) may also develop on heels in orthostatism and at wrists after compression. Reduced connective tissue stiffness at abdominal fascia facilitates formation of inguinal, crural, umbilical, and epigastric hernias, especially in conjunction with increased intra-abdominal pressure (e.g., obesity, pregnancy). More rarely, small muscle herniations may form at sites of discrete areas of incontinent perimysium and be equally visible at examination. Velvety and soft skin is a further common skin texture change. Skin fragility causes increased tendency to skin lesions and lacerations, but such a feature is rarely of concern in JHS/EDS-HT. Keratosis pilaris seems more common in JHS/EDS-HT, but no systematic study assessing such an association has been carried out. 

Minor wound healing defects and capillary fragility are further common features in JHS/EDS-HT. The former may present as atrophic, nonpapyraceous scars, compared to the “cigarette-paper” and crumpled scars observed in other EDSs. They are the consequence of minimally delayed wound repair combined with skin fragility at sites exposed to repeated traumas, such as knees and elbows. The dystrophic nature of such scars may be easily demonstrated by gentle squeezing between observer's fingers. Occasionally, defective wound healing after surgery or extensive/profound traumas may hesitate in full thickness dermal atrophy with consequent subcutaneous fat herniations with anetoderma-like features (i.e., incisional hernia). Capillary fragility causes increased tendency to and delayed resolution of ecchymoses. Spontaneous subcutaneous and intramuscular hematomas are possible rare complications of deep vessels fragility. Finally, many patients suffer from perturbed perspiration in form of diaphoresis or hypohidrosis [65]. In my experience, palmoplantar hyperhidrosis with body hypohidrosis is the most common presentation of exocrine dysregulation. This phenomenon may be neurogenic in origin and relate to the underlying dysautonomia (see Section 5.6). 

### 5.2. Mucosal and Oropharyngeal Features (Figure 2)

Mucosal involvement is common in JHS/EDS-HT. Xerostomia, xerophthalmia, and vaginal dryness are frequent accompanying complaints. Together with hypohidrosis, mucosal xerosis could be a remote consequence of autonomic dysregulation [65, 66]. Blue sclerae are overrepresented among JHS/EDS-HT patients and are likely caused by more visible uveal blood vessels through thinner sclerae [65]. Focal blue-purple discolorations of the oral mucosa are not uncommon in JHS/EDS-HT, and their origin may parallel blue sclerae. Minor pigmentation anomalies of the enamel can be observed in JHS/EDS-HT, also in the absence of environmental causes (e.g., smoking).

Increased mucosal fragility can lead to spontaneous epistaxis and, more commonly, gingival bleeding, which is often elicited by teeth brushing. Repeated gingival damage due to increased mucosal fragility may progressively cause recurrent gingival inflammations/infections, gingival retractions, and, eventually (although rarely), true parodontopathy with premature tooth loss [67]. Impaired oral cleanliness related to increased gingival bleeding and restraint of wrist and finger mobility may be the cause of the higher rate of caries in JHS/EDS-HT [67].

In the last decade, attention has been posed on the absence of the lingual and inferior labial frenulum in EDSs [68]. Subsequent reports offered contrasting results [69–72]. More recently, a functional origin for the apparent agenesis/absence of the lingual frenulum in JHS/EDS-HT has been emphasized. In fact, this feature is likely the results of multiple contributors, such as primitive (developmental) hypoplasia of the frenulum and uncoordinated tongue movements due to concomitant orofacial dyspraxia [73]. Although still unsupported by evidence-based investigations, oropharyngeal dysphagia seems common in JHS/EDS-HT and, in rare instances, may impede feeding with consequent excessive weight loss, exacerbation of fatigue, and, in children, failure to thrive. 

Temporomandibular joint (TMJ) dysfunction is reported in >70% of JHS/EDS-HT patients [74, 75]. It is partly determined by TMJ hypermobility, as documented by increased mouth opening (i.e., mandibular depression over 50 mm in adults) and voluntary subluxations in asymptomatic subjects. Over the years, TMJ hypermobility becomes complicated by clicks, arthralgias, myofascial pain, masticatory dysfunction, and, eventually, articular locks. Similarly to other joints, it is likely that a primary lack of coordination (dyspraxia) of the masticatory muscles may cooperate with JHM in determining dysfunction. 

### 5.3. Orthopedic Features (Figure 3)

Congenital capsulo-ligamentous laxity (CLL) is the primary articular feature of JHS/EDS-HT. In both physiologic and pathologic conditions, it may determine excessive joint motion. JHM is the clinical consequence of increased joint mobility along physiologic axis(es) and may be measured by comparison with standards. Meanwhile, joint instability is used to define the capacity of a lax joint to move along nonphysiologic axes. Both contribute in generating the various EDS orthopedic complications, which comprise increased tendency to (sub)luxations, sprains, and soft-tissue lesions (e.g., bursitis, tendonitis, synovitis, tenosynovitis, and fasciitis) [13]. While, in some patients, repeated dislocations/sprains may further weaken capsuloligamentous resistance and, thus, worsen joint instability, in others, progressive joint stiffness may progressively limit such a phenomenon. 

Generalized CLL also influences the late stage of morphogenesis, which starts during fetal life and extends much beyond birth. Mechanical stimuli, such as gravity, uterine constraint, and muscle contractions, on growth and molding of the skeleton are likely more effective in a body with lax joints. For this reason, a series of orthopedic dysmorphisms and minor variants usually converge in the JHS/EDS-HT patient and often depict a recognizable pattern (Table 5). 

Precocious osteoarthritis, spondylosis, and lower bone mass are potential degenerative complications of generalized CLL, and all are commonly encountered in JHS/EDS-HT. However, at the moment, there are some concerns on the protective rather than predisposing factor for osteoarthritis of JHM [76], while some preliminary studies pointed out the possibility of a higher rate of osteopenia and osteoporosis among JHS/EDS-HT patients [77, 78]. Further studies urge in order to better define such relationships and identify more reliable assessment and therapeutic protocols. 

Noncanonic interpretation of the effects of generalized JHM on heath and disease anticipates the existence of a wide variety of functional, developmental, and degenerative consequences, which show unexpectedly intimate and multidimensional relationships with disability and quality of life. 

### 5.4. Neurologic Features

Neurologic implications of JHS/EDS-HT have been largely ignored in the past. More recently, much attention has been posed on nervous system involvement, as it has been recognized as a major contributor to disability in EDS [6]. In 2009, Voermans and colleagues pointed out a possible JHS/EDS-HT neurologic phenotype characterized by a high rate of myopathic electrophysiologic findings possibly combined with reduced sensation and muscle weakness, increased muscle echo intensity, and myopathic changes at biopsy [79]. Previous studies highlighted an association with neuropathies [80–82] and myalgias with cramps [83]. 

Chronic/recurrent pain and fatigue are, by far, the most common neurologic complaints, being reported in many EDS patients and in, perhaps, all adults with JHS/EDS-HT [84, 85]. Pain manifestations are widespread and involve the musculoskeletal system, as well as the nervous system and internal organs (Table 6) [19, 47, 53, 54, 67, 75, 84, 86–93]. Their origin is largely obscure except for a statistical association between limb/joint pain and (i) regular analgesic use, (ii) JHM, (iii) dislocations, and (iv) previous surgery [84]. Multiple studies demonstrated that chronic fatigue is a major contributor to disability in JHS/EDS-HT [85, 94, 95]. Associated complaints include muscle weakness [96], sleep disturbance [97], and other features of chronic fatigue syndrome [95]. 

Headache is a highly disabling form of pain in EDS [87, 90]. In JHS/EDS-HT, migraine seems the most common form of headache [98]. However, JHM, especially in form of cervical spine instability, is a possible trigger for other headache disorders, including new daily persistent headache [60], cervicogenic headache [99], and neck-tongue syndrome [100]. Headache attributed to spontaneous (idiopathic) cerebrospinal fluid leakage is a further form of headache possibly facilitated by connective tissue laxity and then associated with JHM [57]. Recently, postsurgical recurrence of Chiari I malformation and associated symptoms, also comprising headache, emerged as a predictor for an underlying heritable connective tissue disorder [40]. Taken together, these evidences may explain the extreme clinical heterogeneity, treatment resistance, and high impact on quality of life of headache in JHS/EDS-HT. 

Consolidated evidence indicates that JHS associates with impaired proprioception at various joints, such as proximal interphalangeal joints [101] and knee [102], with consequent poorer joint kinesthesia and position sense [103]. Such a lack of proprioception, that likely affects multiple body segments, impairs balance and posture [104–107]. Clumsiness, tendency to falls, and fear of falling are direct consequences of this phenomenon [108]. The likely congenital nature of such a proprioception impairment may contribute to delayed autonomous walking, tip-toe walking, lack of crawling, clumsiness, and, possibly, dyspraxia, which are frequently reported in infancy and childhood by JHS/EDS-HT patients [109]. For this reason, a noncasual association between JHS/EDS-HT and developmental coordination disorder has been recently proposed [49]. 

### 5.5. Psychiatric Features

While emotional/behavioral distress is quite common in various EDSs and significantly contribute to disability, the relevance of psychologic/psychiatric features and their likely relationships with the underlying pathophysiology are generally overlooked in the management of these patients. By studying 48 EDS patients (including eleven with a clinical diagnosis of EDS-HT and five with JHS), Lumley et al. detected a high rate of anxiety, depression, anger, and interpersonal concerns [110]. Interestingly, access to psychiatric services was registered in ~2/3 patients. A considerable excess of emotional symptoms [111] and psychological distress and somatosensory amplification [38] are noted in JHS/EDS-HT patients. More specifically, JHS/EDS-HT is more common among patients suffering from anxiety and panic disorders and, in turn, these complaints are frequently reported in JHS/EDS-HT [112, 113]. Although psychological difficulties may be secondary to chronic pain and disability, ostracism, and avoidance of relationships, a primary (i.e., pleiotropic) and/or organic contributor may coexist. Accordingly, Eccles et al. described greater amygdale volumes in reportedly hypermobile compared with nonhypermobile subjects [114]. Additional findings included decreased volume of anterior cingulate and parietal lobe. Volumetrically abnormal regions are implicated in cognitive control of pain and negative emotions [115]. It is well known that behavior is influenced by the environment, via neural afferents, as an adaptive reply to the homeostatic need. Reactive behavior changes induce, in turn, autonomic arousal states which translate in action such a reply. Therefore, in JHS/EDS-HT, it is possible that, in the future, some behavioral/psychological characteristics could be unexpectedly linked to specific functional features, such as dysautonomia and lack of proprioception. 

### 5.6. Cardiovascular and Pulmonary Features

Cardiovascular involvement is a feature of many HCTDs, including JHS/EDS-HT. Mild mitral, tricuspid, and aortic valve regurgitation is observed in ~25% patients with classic EDS or EDS-HT [119]. However, true mitral valve prolapse occurs in ~6% patients only [120, 121], and this incidence does not seem significantly higher than controls [120]. Early investigations pointed out a high rate of aortic root dilatation in EDS-HT with risk of possible life-threatening complications [122]. A subsequent study on 252 patients with classic EDS and EDS-HT fixed to 10.8% the overall incidence of aortic root dilatation in these conditions, with the latter showing the highest risk (12%) [121]. Of note, at variance with other HCTDs with reduced life span, in all but one patient aortic dilatation did not show any progression in adulthood. 

Besides such minor structural heart anomalies, dysautonomia is, by far, the most clinically relevant cardiovascular feature in JHS/EDS-HT. Rowe et al. described 12 EDS patients (six with classic EDS, six with JHS/EDS-HT) with orthostatic intolerance demonstrated by orthostatic stress test [123]. Subsequent clinical and experimental studies drew attention to dysautonomia as a likely underlying mechanism for various visceral complaints in JHS/EDS-HT [91, 124]. More recently, postural tachycardia syndrome has been defined as the most specific form of cardiovascular autonomic dysfunction in JHS/EDS-HT [62].

Morgan et al. found an increased rate of asthmatic symptoms and atopy associated with increased lung volumes, impaired gas exchange, and an increased tendency of both the lower and upper airways to collapse in JHS/EDS-HT [125]. Soyucen and Esen postulated that JHS/EDS-HT may predispose to asthma [126]. In fact, JHS/EDS-HT may lead to persistent childhood wheezing by causing airway collapse through a connective tissue defect affecting airways structure [126]. Further studies are needed to confirm this hypothesis. 

### 5.7. Ocular Features

As previously described, blepharochalasis, antimongoloid palpebral slant, and blue sclerae are relatively common findings in JHS/EDS-HT [65, 127]. Further, though less common features include myopia, unilateral ptosis, and tilted optic disc [127]. A recent survey on 22 patients defined the JHS/EDS-HT phenotype as mostly consisting in xerophthalmia (i.e., positive BUT and Schirmer I tests), steeper corneas, pathologic myopia, and minor lens opacities and vitreal abnormalities [66]. Overall, ocular complaints are usually mild to moderate in JHS/EDS-HT and only a minority of them (i.e., xerophthalmia, and pathologic myopia) need treatment and monitoring, which, at the moment, can be carried out following standard procedures. As some of these features are quantitative and less influenced by age compared to JHM, it is expected that a more accurate ophthalmologic assessment will be included in a revised version of JHS/EDS-HT diagnostic criteria. 

### 5.8. Gastrointestinal Features

Although not included in the Villefranche and Brighton criteria, gastrointestinal involvement is common in JHS/EDS-HT. Indirect evidence comes from several studies demonstrating a high incidence of JHS or (generalized) JHM among patients suffering from chronic (slow transit) constipation [41–43], hiatus hernia [58], Crohn's disease [48], faecal incontinence [50], rectal evacuatory dysfunction [63], and functional gastrointestinal disorder [56]. Typical gastrointestinal features include gastroesophageal reflux (74%) with or without hiatus hernia, chronic/recurrent gastritis (48%), symptoms of delayed gastric emptying, recurrent abdominal pain (68%), and constipation/diarrhea (72%) [19]. However, the range of bowel involvement may extend much beyond to include a wide variety of functional gastrointestinal disorders according to the Rome III classification [128]. 

**Figure 4:**
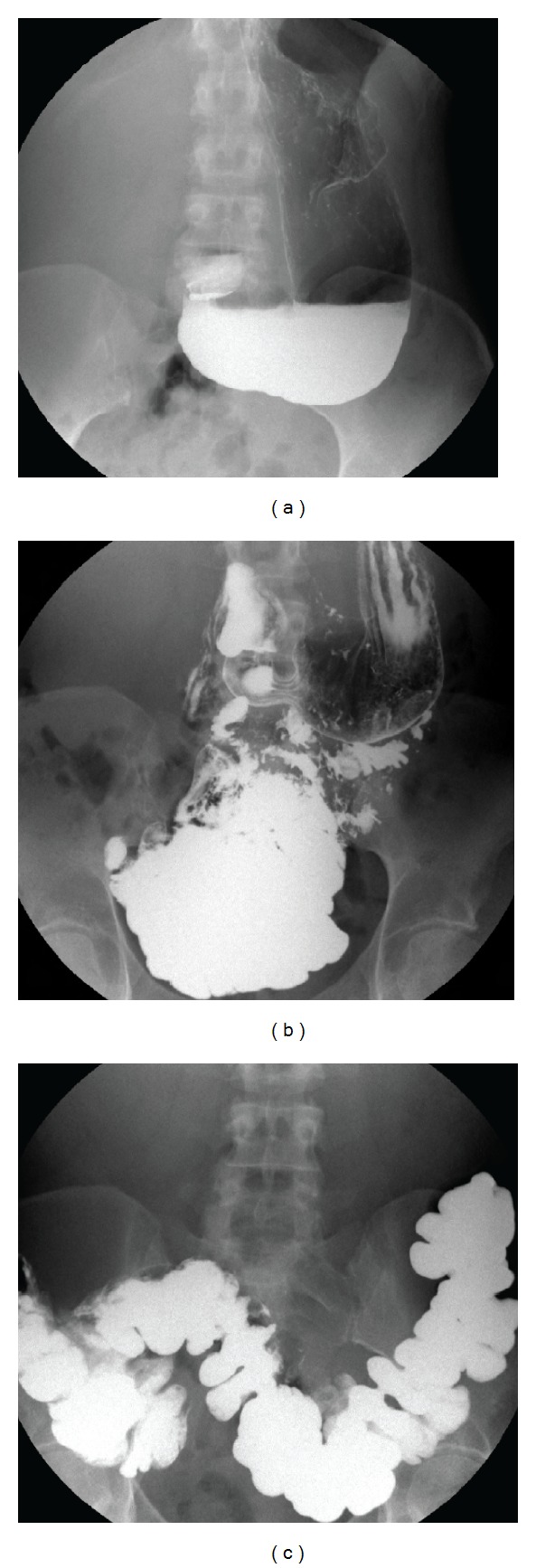
Visceroptosis of the gut in a 40-year-old woman with severely debilitating gastrointestinal functional complaints. Note marked gastroptosis (a) and pelvic localization of the small bowel (b) and transverse colon (c).

The mechanisms underlying such a severe visceral involvement are obscure. Possible contributing factors may comprise (i) reduced fixation to adjacent structures causing visceroptosis and hernias, (ii) gut hypotonia/hypomotility, and (iii) structural anomalies (e.g., dolichocolon). A recent study demonstrating an increased rate of celiac disease in JHS/EDS-HT [129] adds complexity to the study of connections between connective tissue and bowel function, which appear also mediated by an abnormally functioning immune system. The apparent underdiagnosis of visceroptosis in JHS/EDS-HT has been recently pointed out [130]. Accordingly, while literature data concerning such a disease manifestation is scarce, clinical practice often reveals abnormal downward displacement of the gastrointestinal tract and kidneys (Figure 4). The impact of such anatomic features, as well as gut motility and length, in symptom development needs further clarification. 

### 5.9. Gynecologic Features

Gynecologic aspects of JHS/EDS-HT have been largely ignored in the past. However, it is now clear that women with JHS/EDS-HT commonly suffer from irregular menses, meno/metrorrhagias, and severe dysmenorrhea [92]. The latter may only occasionally be related to an underlying gynecologic disorder, such as endometriosis, and, therefore, displays a (dys)functional origin in most cases. Fertility and pregnancy are usually unaffected by JHS/EDS-HT, although more attention should be posed on obstetric and anesthetic interventions in order to prevent some potentially life-threatening or disabling complications. Among them there are (i) the risk of anesthesia-induced hypotension facilitated by dysautonomia, (ii) meningeal fragility complicating in cerebrospinal fluid hypotension in case of epi/peridural anesthesia, (iii) proneness to pelvic prolapse after episiotomy, and (iv) an apparently increased rate of suture dehiscence and minor hemorrhages after surgery. Although, in the past, previous case reports or case series gathering data from different EDS forms suggested prudence in counseling pregnancy in this condition [131–140], recent data are more reassuring. Associated symptoms are influenced by pregnancy. But, while symptoms worsen in many cases, in other women they remain unchanged or improve during pregnancy [92]. 

Pelvic prolapse is the most debilitating gynecologic feature of JHS/EDS-HT, and, accordingly, it was comprised in the revised Brighton criteria [9]. Clinical manifestations mainly include urinary stress incontinence [64], uterine prolapse, and faecal incontinence [50]. Although prolapses may occur in the nullipara [141], they are most often facilitated by episiotomy and vaginal tears [92]. 

## 6. Disease Evolution

As recently outlined, JHS/EDS-HT displays marked phenotypic metatropism with extreme variability at various ages [19, 141]. A series of mechanisms may explain such a phenomenon. Firstly, excessive joint motion is not always detrimental and it often precedes by some years or decades of musculoskeletal pain. At the same time, JHM naturally decreases with age also in the prospectively symptomatic patient. This implies that many patients refer to the general practitioner or specialists when JHM is no more visible, at least, at Beighton score calculation. Secondly, as introduced by the Brighton criteria [9] and the modified Villefranche criteria [17], JHS/EDS-HT manifestations extend much beyond the musculoskeletal system and many disabling features progress uncoupled with joint motion. Thirdly, many patients develop a series of avoiding strategies, such as kinesiophobia, with the false hope of reducing disability. The consequence of such maladaptive cognitions is a progressive limitation of daily activity with aggravation of muscle deconditioning, and, eventually, musculoskeletal pain and fatigue [142]. The understanding of these processes allows to identify at least three distinct disease phases, whose knowledge may help physicians in suspecting JHS/EDS-HT at various ages. For details on the disease progress and phases delineation, see [19].

In addition to the postnatal progression of the disease, connective tissue fragility may also affect resilience of the membranes and cervix, as well as late fetal development. The neonatal presentation of JHS/EDS-HT may comprise slightly preterm birth, precipitous delivery, congenital dislocations at shoulders and clavicles, congenital hip dislocation (usually, unilateral), clubfoot, and, possibly, positional plagiocephaly. Presence of multiple features at birth should lead to investigate JHS/EDS-HT or apparently asymptomatic JHM (either visible or historical) in one of the parents, who may have transmitted the trait. Conversely, neonatal hypotonia, congenital scoliosis, dislocations at unusual sites (e.g., knee or occipitoatlantoaxial junction), fractures, and skin lacerations are never been reported in JHS/EDS-HT and are more typical of other HCTDs. 

## 7. Diagnosis

To date, the diagnosis of JHS/EDS-HT is clinical in essence and based on the agreement of largely accepted diagnostic criteria together with the exclusion of other, partially overlapping HCTDs. Assessment of JHS/EDS-HT lays exclusively on accurate clinical history taking and extensive physical examination, including dermatologic, oral cavity, orthopedic, and neurologic evaluations (see Section 5). However, skin and joint motion assessment deserves annotations. 

### 7.1. Skin Extensibility

Skin extensibility is difficult to assess due to the lack of standardized methodologies. Over the years, a series of tools have been proposed [143–145] but none of them is used in the clinical practice. More recently, skin extensibility measurement by using a suction cup has been proposed as a reproducible method [146]. However, a the moment, skin extensibility evaluation is largely left to the practitioner's experience. As a rule of thumb, skin is considered “hyperextensible” in an adult if it can be stretched by ≥1.5–2 cm at the dorsum of the hand (fourth metacarpal), and/or volar aspect of the forearm. Testing in areas of natural skin redundancy (i.e., extensor surfaces) should be avoided. Similar parameters are not available for measuring velvety/smooth skin. Assessing skin consistency and texture is harder in toddlers and infants due to their inherent softness of tissues. 

### 7.2. Joint Mobility

**Figure 5:**
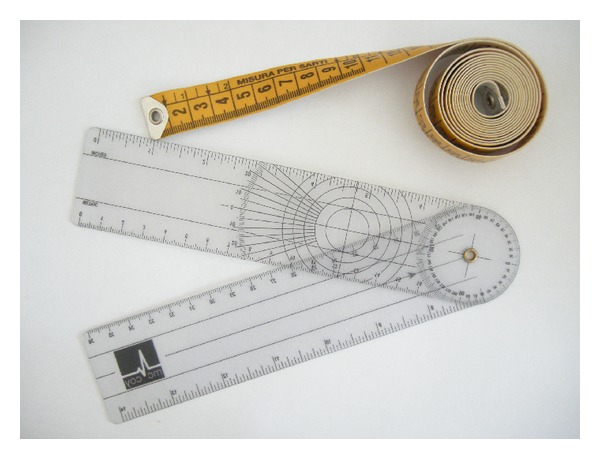
Illustrative examples of universal goniometer and flexible tape as essential tools for assessing joint mobility.

In most joints, evaluation of range of motion is obtained by manipulation and is more accurate if supported by an anatomic (universal) goniometer, a ruler and/or a flexible tape (Figure 5). Various systems are available for identifying subjects with generalized JHM, and the Beighton score is the most widely accepted one (Table 7) [10]. Application of this score is not ideal in all situations. Firstly, specific subpopulations, such as females and pre-pubertal individuals, are inherently more “lax” than others. Secondly, many joints and groups of joints are not considered in the computation. Thirdly, JHM naturally decreases with age also in the affected/symptomatic individuals. Therefore, specific and/or disabling symptoms may set up or worsen when JHM is no further appreciable. In order to investigate JHM in joints other than those included in the Beighton score, standards are available for adult (i.e., ≥16 years of age) subjects (Table 8) [147]. Similarly, a set of specific questions has been outlined for detecting historical JHM in subjects who lost their double jointedness (Table 9) [117]. Similarly to skin extensibility, objective evaluation of variability in joint motion is difficult in toddlers and infants, as the Beighton score is nearly useless in these subjects and alternative screening tools are not yet available. 

### 7.3. Ultrastructural and Molecular Findings

At the moment, ultrastructural and molecular abnormalities are only occasionally identified in JHS/EDS-HT and no finding is pathognomonic of this condition. Therefore, skin biopsy and molecular testing do not take part to the standard diagnostic schedule in JHS/EDS-HT. In the past, the ultrastructural features of EDS-HT have been studied and their knowledge may be relevant, as such investigations can be performed for the differential in doubtful cases. In particular, early studies showed that single collagen fibers of the reticular dermis may present an enlarged and irregular section, but usually without coalescing in the typical “cauliflower-like” fibers observed in classic EDS [148]. Molecular analysis of *TNXB* and *COL3A1* is not confirmatory for JHS/EDS-HT, although it may be considered for differential diagnosis in case of partial overlap with the vascular and Tenascin X-deficient forms of EDS. 

### 7.4. Diagnostic Criteria

JHS/EDS-HT is an exclusion diagnosis based on published diagnostic criteria. Actually, two distinct sets of diagnostic criteria exist: the Villefranche criteria for EDS-HT [4] and the Brighton criteria for JHS [9]. The Villefranche criteria emerged from the activities of an international group of experts mainly comprising pediatricians and clinical/medical geneticists and are typically used to evaluate children and young adults. Independently, JHS, alternatively termed hypermobility syndrome, generalized joint hypermobility syndrome, or benign joint hypermobility syndrome (the latter is now in disuse as JHS should be no longer considered a “benign” condition), has been studied by the rheumatologic community. The Brighton criteria consider the natural progressive loss of joint mobility by age and, therefore, are more adequate to assess adults. Consequently, in order to be considered affected by JHS/EDS-HT, a patient must meet either the Villefranche or the Brighton criteria. A further set of diagnostic criteria was proposed by Levy and was derived from the Villefranche criteria for EDS-HT [17]. It includes additional common features not previously considered, such as functional bowel disorder and cardiovascular dysautonomia, and, then, seems more inclusive. However, it has not yet been validated by the scientific community. In light of the recent discoveries concerning the protean manifestations of JHS/EDS-HT, the need of extensively revising and unifying available diagnostic criteria for this condition is urgent [149].

### 7.5. Differential Diagnosis

The extreme clinical variability of JHS/EDS-HT identifies a great number of partially overlapping (acquired and genetic) disorders showing the variable association of mucocutaneous fragility, JHM, chronic musculoskeletal pain and fatigue. Among them, there are other HCTDs with JHM, the “battered child” syndrome, bleeding disorders, and various rheumatologic conditions with chronic musculoskeletal pain, such as ankylosing spondylitis, rheumatoid arthritis, and fibromyalgia. The association of apparently unexplained features (see “clinical features”) and severe physical disability observed in JHS/EDS-HT broadens the spectrum of the differential to include some neurologic disorders, including multiple sclerosis, amyotrophic lateral sclerosis, hereditary and acquired sensory-motor and/or autonomic polyneuropathies, and chronic fatigue syndrome, as well as myopathies featuring JHM [30]. Accordingly, the number of possibly useful investigations is myriads. Their use should be wisely evaluated case by case, as no standardized guidelines are available up to date. In the clinical practice, many JHS/EDS-HT patients reach the correct diagnosis after dozens of consultations, as well as costly and invasive/ineffective diagnostic procedures. Doubtful clinical pictures could be comprehensively investigated by screening for peripheral polyarthralgias (i.e., hands/feet X-rays, HLA-B27 testing, erythrocyte sedimentation rate, and rheumatoid factor, and C-reactive protein dosage) [150], chronic fatigue syndrome panel screening [151], serum creatine kinase and lactate dehydrogenase dosage, and electroneurography/electromyography. 

Among the various HCTDs sharing some musculoskeletal features with JHS/EDS-HT, there are other EDSs, as well as the Loeys-Dietz and arterial tortuosity syndromes. Historical and physical clues for suspecting such conditions include papyraceous/hemosiderotic scars, molluscoid pseudotumors, subcutaneous spheroids, markedly visible subcutaneous vessels, triangular face with sunken eyes, early-onset cutis laxa and premature ageing, bifid uvula/cleft soft palate, hypertelorism, dolichocephaly, vascular complications, history of osteochondritis dissecans, and sudden death in close relatives. Persistence of the suspect of vascular EDS, Loeys-Dietz, or arterial tortuosity syndromes should be further investigated by extensive vascular imaging (i.e., whole-body angio-MRI; or brain angio-MRI plus thoracic and abdominal angio-TC; or heart, abdominal aorta, epiaortic and limb vessels Doppler ultrasound) followed by molecular testing (*COL3A1*, *TGFBR1*, *TGFBR2*, *SMAD3*, *TGFB2,* and *SLC2A10*) in specialized laboratories. Rapid detection of such rarer HCTDs is crucial for prognosis establishment due to their high risk of vascular accidents, a complication never reported in JHS/EDS-HT. Occasionally, differential diagnosis with classic EDS may need *COL5A1* and *COL5A2* molecular testing. 

## 8. Principles of Management

Guidelines for managing JHS/EDS-HT are still lacking. General indications for the broader group of EDSs have been recently revised and proposed [152], but no program is available for JHS/EDS-HT up to date. Levy presented some suggestions specifically addressed to JHS/EDS-HT [17]. Here, previously published and author's personal experiences are summarized in order to present a structure for approaching treatment and prevention strategies in JHS/EDS-HT. It has not been emphasized enough that, at the moment, the long-term treatment of JHS/EDS-HT is largely unsuccessful in terms of amelioration of symptoms. In fact, after years of treatment cycles and follow-up evaluations, many patients still refer the complaints reported at first evaluation. This anticipates that, actually, the best result of all practitioners' efforts is to stabilize symptoms with short periods of complete/partial relief. It should be taken in mind that most of the following indications are not yet been confirmed by evidence. 

### 8.1. Treatment of Pain: An Overview

Musculoskeletal pain is a major determinant for deterioration of quality of life in JHS/EDS-HT. Although it usually starts as occasional/recurrent joint pain facilitated/triggered by joint instability (e.g., dislocations and sprains), subsequently it becomes pathogenically heterogeneous usually manifesting in form of widespread myalgias and arthralgias and often with neuropathic features. Pain chronicization and resistance to treatment are the most relevant features influencing prognosis. The best management program should include drugs, physical therapy [88, 153, 154], cognitive-behavioral therapy [142], and adherence to a series of lifestyle recommendations [118]. For this reason, while occasional and low-to-moderate recurrent pain may be treated in an outpatient setting by the reference specialist (e.g., clinical geneticist, rheumatologist, physiatrist, or general practitioner), management of chronic or highly disabling recurrent musculoskeletal pain in JHS/EDS-HT usually needs a multidisciplinary approach. 

### 8.2. Treatment of Pain: Medications

In my experience, drugs monotherapy is successful for treating occasional/recurrent pain of low-moderate intensity. The following alternatives are of potential use in the otherwise healthy adult with JHS/EDS-HT (adjustments are needed for children and nonhealthy subjects) and can be well managed by the general practitioner:

1. ibuprofen 200–1,800 mg/day (mean: 1,200 mg/day) in one to three divided doses with a maximum of single dose of 600 mg;
2. naproxen 1,000 mg/day in two divided doses;
3. paracetamol 1,000–3,000 mg/day in up to three divided doses of 500–1,000 mg each; paracetamol can be administered in association with codeine phosphate (with a ratio of 30 mg codeine phosphate per 500 mg paracetamol) for a maximum daily dose of 3,000 mg for the former and 180 mg for the latter.

In case of inefficacy of monotherapy, other drugs may be used in alternative of or association with the above-mentioned medications:

1. tramadol up to 400 mg/day in one of two divided doses with a maximum single dose of 200 mg; association of codeine phosphate and tramadol must be avoided;
2. Cox-2 inhibitor (celecoxib) 200–400 mg in one or two divided doses in presence of documented osteoarthritis;
3. pain modulator drugs, including tricyclic antidepressants and serotonin/norepinephrine receptor inhibitors, in presence of documented or presumed neuropathic pain; among them, amytriptyline is actually considered the best choice [142] with an initial daily dose of 10 mg with an increase of 10 mg/week (up to 100/day) after careful monitoring of pain relief and side effects (the preferred dose is usually 30–50 mg/day); duloxetine is a further promising drug; benefits from these drugs also include treatment of other satellite symptoms, such as depression, sleep disturbances, and irritable bowel disease;
4. opioids (other than tramadol and codeine) may be effective for treating chronic musculoskeletal pain of moderate-to-high intensity, and their management usually needs specialist consultation; although the use of such drugs is usually successful in the short terms, many chronic JHS/EDS-HT users of opioids still suffer from intense and debilitating pain.

### 8.3. Treatment of Pain: Nonpharmacologic Resources

In addition to drugs, alternative interventions could be considered in isolation or combined with medications and lifestyle modifications (see “Section 8.6”). They include the following.

1. Referral to a physical therapy specialist is expected for all JHS-EDS-HT patients in order to identify the need for specialized intervention, choose the best suited sport/fitness activity, and educate the patient to “joint” health; extensive information for the physical therapist is summarized in Hakim et al. [155].
2. Cognitive-behavioral therapy is beneficial in all forms of chronic pain, including musculoskeletal and visceral pain as well as headache; therefore, cognitive-behavioral therapy is indicated in patients with debilitating pain not adequately treated by standard care (i.e., probably most painful sensations other than those related to dislocations and sprains).
3. Application of braces for short periods may improve joint stability in case of recurrent sprains and their use needs specialistic consultation;
4. Soft neck collars, waterbeds, adjustable air mattresses, and viscoelastic foam mattresses and/or pillows may improve quality of life related to headache and sleep quality.
5. Crutches, canes, walkers, wheelchairs, and scooters may improve residual mobility in the most disable patients; while their use allows the patient to perform some activities easier, it is not free of side effects, such as joint traumas to the upper limbs and increased deconditioning of the lower limbs.
6. Occupational therapist consultation is usually indicated for pain conditions influencing daily activity and, then, constricting life of the affected individual; it is needed in presence of loss of autonomy and disability at home, work, and school.
7. JHS/EDS-HT pelvic pain is best managed with a multispecialist approach but a specific schedule is lacking; general recommendations are well summarized in Nelson et al. [156].
8. Pain related to gastrointestinal functional disorder can be managed following available standards [157, 158].
9. Topical lidocaine (cream or patch), local injections of anesthetic and steroids, and anesthetic nerve blocks are possible non- or mini-invasive procedures that prevent systemic assumption of drugs and, consequently, the risk of side effects; no systematic study is available, but their use seems of very limited success (see Section 8.7).
10. Recently, prolotherapy with 10% dextrose was demonstrated successful in reducing pressure-induced pain at the TMJ in the hypermobile subject [159]; with caution, its use may be considered in other joints also.
11. Although considered at risk of causing dislocation, chiropractic with application of low-force adjusting techniques may be successful in JHS/EDS-HT [160].
12. Heat and hydrotherapy, acupuncture, and transcutaneous electrical nerve stimulation (i.e., TENS) are possible alternatives without overt contraindications in JHS/EDS-HT; their effectiveness remains untested.

### 8.4. Treatment of Pain: Points of Concern

Some therapeutic options display documented/presumed severe side effects or hopeless inefficacy and, then, should be considered most cautiously. Among them there are the following.

1. Most of the orthopedic surgical interventions aimed at stabilizing joints, such as arthroscopic debridement, tendon relocations, capsulorrhaphy and arthroplasty, and reducing annulus hernias (e.g., high risk for recurrence, abnormal wound healing, adhesion formation, and pain amplification); surgery should be always postponed to more conservative approaches; when it is firmly requested, meticulous planning and communication to the patient of the low rate of success of this approach are mandatory.
2. Generous prescription of periods of inactivity and abstention from regular sport activity (i.e., muscle deconditioning of rapid onset).
3. Use of myorelaxants (i.e., amplification of joint instability with multiple dislocations with consequent exacerbation of pain and fatigue).
4. Chronic local and systemic use of steroids (i.e., steroid-induced connective tissue damage on soft-tissues and bone).
5. Use of antiplatelet drugs, for example, as acetylsalicylic acid (i.e., increased tendency to mucosal hemorrhages and ecchymoses).
6. Use of antiepileptic drugs (i.e., exacerbation of dysautonomic symptoms).

### 8.5. Treatment of Fatigue

In JHS/EDS-HT chronic fatigue is likely multifactorial and pathogenic heterogeneous [118]. Although various contributors, such as muscle weakness, cardiovascular dysautonomia, sleep disturbance, malabsorption, respiratory dysfunction, and analgesic overuse, may be clinically identified and, possibly, managed, no study has been performed to systematically investigate these factors weighting their role isolatedly and possible treatments. The above-mentioned factors may influence the general wellness of affected individuals and all or most of them should be properly investigated in any JHS/EDS-HT patient displaying a disabling fatigue. Distinguishing between physiologic fatigue after physical activity or due to unhealthy lifestyle and pathologic fatigue may be difficult. However, the coexistence of persistent exertional dyspnea, unrefreshing sleep, postexertional malaise, and reduced stamina, combined with major limitations of the daily activities, likely indicates pathologic fatigue. Common comorbidities, most of which concurring by chance, should be properly investigated and treated [151]. Sleep hygiene, gastroenterologist and pneumologist consultation, and drug therapy adjustments are strategies which may alleviate fatigue-related disability. Nevertheless, cardiovascular dysautonomia seems the most relevant contributor to fatigue in JHS/EDS-HT [62].

Management of chronic fatigue and cardiovascular dysautonomia is firstly nonpharmacological, and life-style recommendations are summarized here, in part (see Section 8.6), and in Mathias et al. [62]. In patients in whom these procedures are ineffective, drug use could be considered. Fludrocortisone in a daily dose of no more than 300 *μ*g is the first-line drug. Vasoconstrictors, mainly midodrine (at an initial dose of 2.5 mg/die which may be weekly raised up to 30 mg/day), are second-line alternatives and could represent a preferred choice in JHS/EDS-HT in consideration of the increased risk of osteopenia/porosis. Both fludrocortisone and vasoconstrictors are contraindicated in patients with systemic hypertension. In this case, *β*-blockers or clonidine may improve both the blood pressure and heart rate. *β*-blockers should be avoided in patients with asthma, a feature with a possibly increased rate in JHS/EDS-HT [126]. In patients with marked postprandial tiredness, octreotide at low dose (25–50 *μ*g in three administrations before the principal meals) is a therapeutic option. Recently, Kanjwal et al. [161] identified modafinil as a possible therapeutic resource for managing chronic fatigue in orthostatic intolerance. For more details on management of pain and fatigue in JHS/EDS-HT, refer to [118]. 

### 8.6. Lifestyle and Nutritional Recommendations

In consideration of the chronic and progressive nature of JHS/EDS-HT and the nonexistence of decisive treatments, adherence to specific behavioral guidelines for preventing symptom onset and/or deterioration appears, at the moment, the most cost- and time-effective solution. Major limits still exist in appropriately selecting and testing reasonable recommendations. This lays down the still too fragmented knowledge on the pathophysiology of JHS/EDS-HT. Nevertheless, a list of likely harmless and potentially effective lifestyle recommendations can be identified (Table 10) [118]. Such suggestions are drawn based on general recommendations for preventing complications related to some common phenotype components of JHS/EDS-HT, such as proneness to joint damage and cardiovascular dysautonomia.

In addition to behavior modifications, adequate nutritional supplementations may be of some help in preventing/treating some features of JHS/EDS-HT. Although specific studies are still lacking, suggestions have been recently proposed [62, 162, 163]. In particular, dysautonomia-related fatigue may be partly managed by (i) generous daily water/liquid intake (2–2.5 lts) preferring isotonic solutions, (ii) high salt intake (to avoid in case of arterial hypertension), and (iii) daily assumption of carnitine (250 mg) and/or coenzyme Q10 (100 mg). Capillary/small vessels fragility may be improved by daily assumption of ascorbic acid, a cofactor of prolyl and lysyl hydroxylases, which are enzymes involved in the biogenesis of collagens. Approximately 8–50 times the 60 mg recommended daily intake for adults is indicated as the dose for maximal improvement of such biological functions. In case of osteopenia, daily intake of therapeutic doses of vitamin D (usually 880 IU/day in adults) and calcium (usually 1,000 mg/day in adults) is indicated for lowering the risk of fracture. Vitamin D is present in a few foods, and many people, especially in USA and Europe, may not get enough sunlight, which is essential for endogenous production of vitamin D from cholesterol. Therefore, a daily supplementation of 200 mg or 400 mg vitamin D, for adults and children, respectively, may be recommended also in the nonosteopenic individual. A 1–5 mg daily intake of melatonin is considered a resource for improving sleepiness in various functional somatic syndromes, such as fibromyalgia. Similarly, melatonin may be effective in JHS/EDS-HT. Other nutraceuticals which have been thought beneficial, though still without evidence, in JHS/EDS-HT comprise vitamin E, vitamin B complex, vitamin K, glucosamine, chondroitin, *γ*-linolenic acid, pycnogenol, magnesium, zinc, methyl sulfonyl methane and silica. 

### 8.7. Surgical and Anesthetic Issues

Major surgical complications, such as organ or vascular rupture, are typical of other EDS subtypes and have never been reported in JHS-HT. Nevertheless, there are some minor weaknesses in JHS/EDS-HT, such as cervical spine and TMJ instability/laxity, delayed wound repair, cardiovascular dysautonomia, and slight vascular fragility, that may be of concern in case of minor or major surgery. Accordingly, some red flags are identified for healthcare professionals approaching surgery in JHS/EDS-HT.

1. Although surgery is not contraindicated in JHS/EDS-HT, the increased time requested for soft-tissue repair and the related risk of possibly unsatisfactory results and muscle deconditioning due to postsurgical recovery entail to pay more attention in planning invasive interventions.
2. The mild soft-tissue fragility and delayed wound healing may be counteracted by doubling the waiting time before suture stitches removal.
3. In case of local/minor surgery, consider the frequently reported resistance to intradermal lidocaine infiltrations and topical EMLA cream in JHS/EDS-HT [164, 165]; a double dose of anesthetic by intradermal injection as the first choice may be effective.
4. Although evidence is lacking, local anesthetic resistance could manifest also in case of epidural anesthesia.
5. Intubation should be performed with care due to TMJ and cervical spine instability and minor mucosal fragility; in adult patients with severe TMJ dysfunction, limited mouth opening may request the use of pediatric devices.
6. Peridural anesthesia administration may request extra time due to premature spondylosis; meningeal fragility may associate with an increased risk of intracranial hypotension due to cerebrospinal fluid leakage.
7. In case of total anesthesia, the coexistence of cardiovascular dysautonomia may increase the risk of hemodynamic changes; prophylactic early fluid loading and phenylephrine infusion should be considered [139].
8. Although postsurgical hemorrhages are usually mild, their occurrence, especially in older subjects and toddlers as well as in case of concurrent chronic diseases, may expose the patient to unreasonable risks; prophylactic use of desmopressin (DDAVP) may be considered to reduce the chance of excessive bleeding.

### 8.8. Obstetric Issues

Besides the risk of disease transmission as previously discussed (see Section 4), the diagnosis of JHS/EDS-HT also has obstetric implications. While fertility does not appear affected by JHS/EDS-HT, particular attention should be posed on delivery planning for both operative and anesthetic implications (see Section 8.7). In addition, in order to minimize the risk of pelvic prolapses, Cesarean section should be considered the first choice when vaginal delivery without episiotomy cannot be anticipated [92]. Slightly preterm delivery due to premature rupture of the membranes or cervical insufficiency and precipitous delivery occur in ~10% and ~30% of cases, respectively, and should be carefully considered. 

## 9. Conclusion: Ehlers-Danlos Syndrome, Hypermobility Type as a Model for Studying Functional Somatic Syndromes

Since its early definition as an HCTD with predominant rheumatologic manifestations, JHS/EDS-HT is emerging as a widespread disorder with reverberations in practically all organs and systems. Although most complications are not life-threatening and many patients have a nearly intact life-span, the pervasive nature of the disorder often makes their life poor and restricted by worsening disability [166]. The spectrum of clinical implications of lax joints even outside rare and well-defined HCTDs seems to be wider than previously expected, in contrast to the quaint adage of considering JHM a benign, asymptomatic trait. Accordingly, Table 4 well illustrates the range of complaints/disorders linked to JHM. Although most studies are based on statistical analysis testing the occurrence of JHM among patients suffering from specific “common” afflictions, these preliminary observations may hide under a common milieau. The existence of such a correlation is foreshadowed by the convergence of most of these complaints in JHS/EDS-HT patients, who often “migrate” from one specialist to another referring every time a different complaint. Accordingly, an underlying disorder of the connective tissue may represent the missing link between JHM and the extra-articular dysfunctions capturing the practitioner's attention also in patients not satisfying the different sets of diagnostic criteria for JHS/EDS-HT.

**Figure 6:**
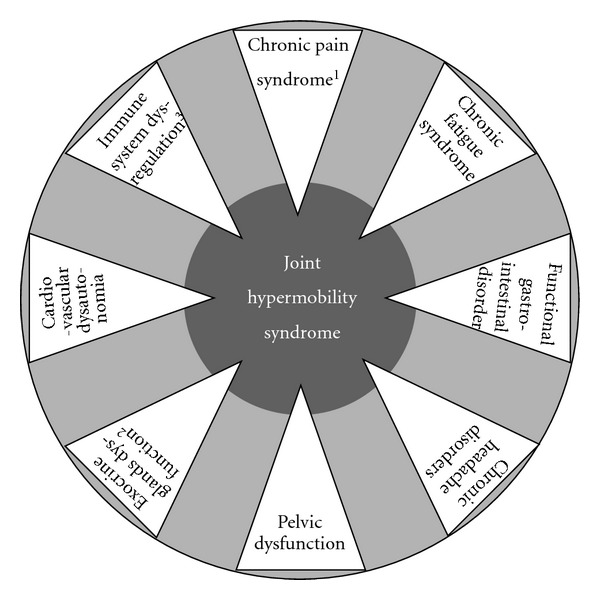
Schematic representation of extra-articular manifestations of Ehlers-Danlos syndrome, hypermobility type (alternatively termed joint hypermobility syndrome). The dark grey circle symbolizes the phenotypic spectrum of this condition, which includes a series of functional somatic syndromes and tissue/organ-specific dysfunctions (i.e., the white triangles, whose tips are indeed comprised within the dark circle). Outside the clinical spectrum of Ehlers-Danlos syndrome, hypermobility type, the single phenotypic components may be observed in isolation or, perhaps, in incomplete associations within the general population (the larger and light grey circle). It is expected that, in the future, the study of heritable dysfunctions of the connective tissue will move from the dark gray circle to the light gray one, as a prominent field of interest. ^1^Mostly including fibromyalgia, myofascial pain and complex regional pain syndromes. ^2^Comprising xerophthalmia, xerostomia, vaginal dryness, and abnormal sweating. ^3^Asthma, atopy, gluten sensitivity, inflammatory bowel disease, and recurrent cystitis are all possible manifestations of an underlying immune system dysregulation.

Such an interpretation extends the horizons of the study of heritable anomalies of the connective tissues to a series of bridging phenotypes filling the gap between true HCTDs and (apparently isolated) functional somatic syndromes, such as fibromyalgia, chronic fatigue syndrome, and functional gastrointestinal disorder(s). In this perspective, JHS/EDS-HT may represent the prototype for testing the complex pathogenic correlations between a primary defect of the connective tissue and disorders of tissues other than skin, joints, and vessels. In fact, clinical practice anticipates a continuum among patients with the full-blown JHS/EDS-HT characteristics and others showing incomplete systemic manifestations (the so-called “overlap” or “borderline” phenotypes) or single organ dysfunctions constituting the JHS/EDS-HT extended phenotype (Figure 6). In this context, the unveiling of JHS/EDS-HT molecular basis and the related pathophysiology could have unexpected effects in understanding and, hopefully, better treating a wide variety of common functional disorders, which actually represent a great challenge for the healthcare system of most industrial countries.

## Figures and Tables

**Figure 2:**
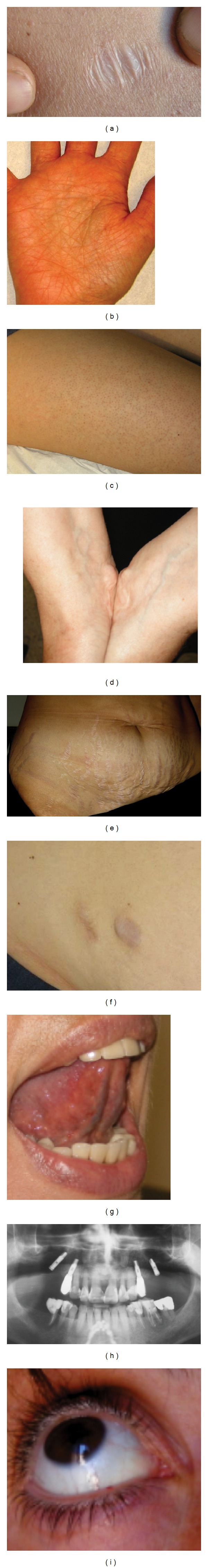
Skin and mucosal features of Ehlers-Danlos syndrome, hypermobility type. Atrophic, nonpapyraceous scar—its atrophic nature is more appreciable after gentle squeezing between examinator's fingers (a). Accentuated crease reticulum of the palm (b). Keratosis pilaris in a 26-year-old woman (c). Piezogenic papules at wrists after compression (d). Extensive abdominal striae atrophicae in a 35-year-old multipara (e). Postsurgical scar with anetoderma-like herniation of the subcutaneous fat (f). Apparent absence of the lingual frenulum (g). Radiographic orthopanoramic showing extensive tooth loss in a 50-year-old man with severe gingival involvement (h). Blue sclerae (i).

**Figure 3:**
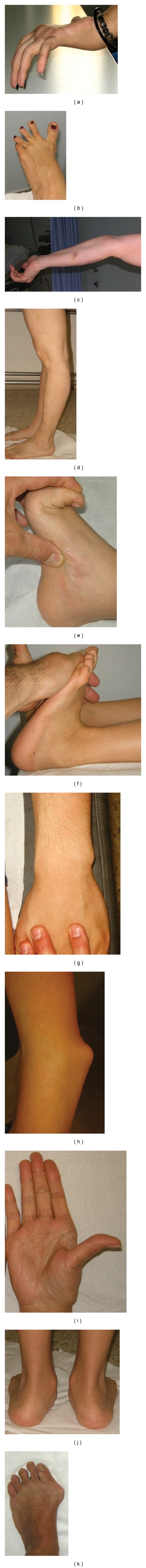
Orthopedic features of Ehlers-Danlos syndrome, hypermobility type. Active joint hypermobility at the fingers (a), toes (b), elbow (c), and knees (genu recurvatum, (d)). Passive hyperextension at great toe (e) and heel (f). Structural changes due to joint instability: fixed subluxation of the distal ulna (g), asymptomatic fixed subluxation of the elbow (h), fixed subluxation of the first metacarpal (i), hindfoot pronation and midfoot eversion in an 11-year-old boy (j), and hallux valgus in a 24-year-old woman (k).

**Table 1:** Classification of EDSs.

Subtype	Inheritance	Gene(s)
Major forms		
Classic	AD	*COL5A1, COL5A2 *
Hypermobility/JHS	AD?	Mostly unknown
Vascular	AD	*COL3A1 *
Kyphoscoliotic	AR	*PLOD1 *
Arthrochalasia	AD	*COL1A1, COL1A2 *
Dermatosparaxis	AR	*ADAMTS2 *
Rare/emerging forms		
Tenascin X deficient	AR, AD?	*TNXB *
Classic with vascular rupture	AD	*COL1A1 *
Cardiac-valvular	AR	*COL1A2 *
EDS/OI overlap	AD	*COL1A1, COL1A2 *
With periventricular heterotopia	XLD	*FMNA *
Musculocontractural	AR	*CHST14 *
Spondylocheirodysplastic	AR	*SLC39A13 *
Progeroid	AR	*B4GALT7 *
Kyphoscoliotic with deafness	AR	*FKBP14 *
Parodontitis	AD	Unknown
Fibronectin deficient	AR	Unknown

AD: autosomal dominant, AR: autosomal recessive, EDS/OI: Ehlers-Danlos syndrome/osteogenesis imperfect, JHS: joint hypermobility syndrome, XLD: X-linked dominant.

**Table 2:** The Villefranche criteria for major EDS subtypes.

Subtype	Major criteria	Minor criteria
Classic	Skin hyperextensibility Widened atrophic scars Joint hypermobility	Smooth, velvety skin Molluscoid pseudotumors Subcutaneous spheroids Complications of joint hypermobility Muscle hypotonia, motor delay Easy bruising Manifestations of tissue extensibility and fragility Surgical complications Positive family history

Hypermobility	Hyperextensible and/or smooth, velvety skin Generalized joint hypermobility	Recurring joint dislocations Chronic joint/limb pain Positive family history

Vascular	Thin, translucent skin Arterial/intestinal/uterine fragility or rupture Extensive bruising Characteristic facial appearance	Acrogeria Hypermobility of small joints Tendon and muscle rupture Talipes equinovarus Early-onset varicose veins Arteriovenous, carotid-cavernous sinus fistula Pneumothorax/pneumohemothorax Gingival recessions Positive family history, sudden death in a close relative

Kyphoscoliotic	Generalized joint hypermobility Congenital hypotonia Congenital and progressive scoliosis Scleral fragility and rupture of the ocular globe	Tissue fragility, including atrophic scars Easy bruising Arterial rupture Marfanoid habitus Microcornea Osteopenia/porosis Positive family history

Arthrochalasis	Generalized joint hypermobility with recurrent subluxations Congenital bilateral hip dislocation	Skin hyperextensibility Tissue fragility, including atrophic scars Easy bruising Hypotonia Kyphoscoliosis Osteopenia/porosis

Dermatosparaxis	Severe skin fragility Sagging, redundant skin	Soft, doughy skin texture Easy bruising Premature rupture of fetal membranes Large hernias (umbilical, inguinal)

Adapted from [4].

Note 1: no clear indication for using these criteria in the establishment of a firm clinical suspect of a specific Ehlers-Danlos syndrome subtype is specified. However, the presence of at least 1 major and 1 minor criteria is usually necessary for proceeding in molecular confirmation of Ehlers-Danlos syndrome subtypes with a known, prevalent molecular cause. The presence of at least two major criteria is strongly indicative for a definite diagnosis of the specific EDS subtype.

**Table 3:** The Brighton criteria for JHS.

The Brighton criteria	
Major criteria	
Beighton score ≥ 4/9	
Arthralgia for >3 months in >4 joints	
Minor criteria	
Beighton score of 1–3	
Arthralgia in 1–3 joints	
History of joint dislocations	
Soft-tissue lesions > 3	
Marfan-like habitus	
Skin striae, hyperextensibility, or scarring	
Eye signs, lid laxity	
History of varicose veins, hernia, visceral prolapse	

Adapted from [9].

Note 1: criteria major 1 and minor 1 are mutually exclusive as are major 2 and minor 2.

Note 2: for the diagnosis of the joint hypermobility syndrome: both major, or 1 major and 2 minor, or 4 minor criteria, or 2 minor criteria and one or more first-degree affected relative(s).

Note 3: diagnosis of joint hypermobility syndrome needs previous (clinical/molecular) exclusion of other overlapping heritable connective tissue disorders, such as Marfan syndrome and other Ehlers-Danlos syndrome subtypes.

**Table 4:** Extra-articular disorders associated with (generalized) JHM.

Condition	Reference(s)
Anxiety	[38, 116]
Carpal tunnel syndrome	[39]
Chiari malformation type I	[40]
Chronic constipation	[41–43]
Chronic fatigue syndrome	[44–46]
Chronic regional pain syndrome	[47]
Crohn's disease	[48]
Developmental coordination disorder	[49]
Faecal incontinence	[50]
Fibromyalgia	[51–54]
Fixed dystonia	[55]
Functional gastrointestinal disorder	[56]
Headache attributed to spontaneous cerebrospinal fluid leakage	[57]
Hiatus hernia	[58]
Mitral valve prolapse	[59]
New daily persistent headache	[60]
Pelvic organ prolapse	[61]
Postural tachycardia syndrome	[62]
Psychological distress	[38]
Rectal evacuatory dysfunction	[63]
Somatosensory amplification	[38]
Urinary stress incontinence	[64]

**Table 5:** Morphologic and orthopedic features of JHS/ED-HT.

Feature	
Leptosomic built or true Marfanoid habitus	
Dorsal hyperkyphosis	
Lumbar hyperlordosis	
Scoliosis of mild degree	
Fixed subluxation of the costochondral and/or sternoclavicular joints	
Fixed dorsal subluxation of the distal radioulnar joint	
Fixed subluxation of the first carpometacarpal joint	
Cubitus valgus	
Femur anteversion^1^	
Patella alta or baja	
Genuum valgum	
Flexible flatfoot	
Hallux valgus	
High-arched/narrow palate	
Facial asymmetry of mild degree (likely secondary to deformational plagiocephaly)	

^1^Intoeing, kissing rotulae, and “W” position of the lower limbs at sitting.

**Table 6:** Forms of pain in EDSs also comprising JHS/EDS-HT.

Pain subtype	Manifestations	Key reference(s)
	Soft-tissue injuries	[86]
	Dislocations	[84]
Nociceptive pain	Arthralgias	[87]
	Back pain	[19, 88]
	Myalgias/myofascial pain	[19, 67, 75]

Neuropathic pain	Compression neuropathy	[89]
	Peripheral neuropathy	[89]

	Complex regional pain syndrome types I and II	[47]
	Fibromyalgia	[53, 54]
Dysfunctional pain	(Some) headache disorders	[90]
	Functional abdominal pain	[19, 91]
	Dysmenorrhea	[92]
	Vulvodynia/dyspareunia	[93]

**Table 7:** The Beighton score for assessing generalized JHM.

Sign	Yes	No
Passive apposition of the right thumb to the flexor aspect of the forearm	1	0
Passive apposition of the left thumb to the flexor aspect of the forearm	1	0
Passive dorsiflexion of the right V finger beyond 90 degrees	1	0
Passive dorsiflexion of the left V finger beyond 90 degrees	1	0
Hyperextension of the right elbow beyond 190 degrees	1	0
Hyperextension of the left elbow beyond 190 degrees	1	0
Hyperextension of the right knee beyond 190 degrees	1	0
Hyperextension of the left knee beyond 190 degrees	1	0
Forward flexion of the trunk with the knees extended and the palms resting flat on the floor	1	0

Adapted from [10].

Note: the Beighton score ranges from 0 to 9. Generalized joint hypermobility is fixed for a total score of 5/9 or above for the Villefranche criteria and 4/9 or above for the Brighton criteria. Unstandardized modifications for specific population subgroups, such as children (*increasing* by 1 point these limits) and males (*reducing* by 1 point these limits), are reasonable. For noncollaborative subjects, a modified Beighton score lacking the spinal bending maneuver and a maximum score of 8 may be applied.

**Table 8:** Standards for evaluating range of motion of adults' joints.

Movement	Maximal ROM
Shoulder elevation through flexion	180°
Elbow extension	190°–195^°1^
Elbow pronation-supination	170^°2^
Wrist flexion	80°
Wrist extension	70°
Wrist ulnar deviation	30°
Wrist radial deviation	20°
2nd finger MCP joint extension	45°
PIP and DIP joint extension	0°
Hip abduction with leg extended	45°
Hip adduction with leg extended	30°
Knee extension	180°–190^°1^
Ankle dorsiflexion	20°
Ankle plantar flexion	50°
1st toe MTP joint extension	70°
Mandible depression	35–50 mm
Mandible protrusion	3–7 mm
Mandible lateral deviation	10–15 mm
Neck rotation	11 cm^3^
Neck flexion	45°
Neck extension	45°
Neck lateral flexion	45°
Thoracolumbar spine lateral flexion	35°

^1^The lower and the upper end fits better for men and women, respectively.

^2^80° in supination and 90° in pronation from mid-position.

^3^From the tip of the chin to the lateral aspect of the acromion process.

DIP: distal interphalangeal, MCP: metacarpophalangeal, MTP: metatarsophalangeal, PIP: proximal interphalangeal, ROM: range of motion.

**Table 9:** A proposed questionnaire for investigating JHM by history.

(1)	Can you now (or could you ever) place your hands flat on the floor without bending your knees?
(2)	Can you now (or could you ever) bend your thumb to touch your forearm?
(3)	As a child did you amuse your friends by contorting your body into strange shapes or could you do the splits?
(4)	As a child or teenager did your shoulder or kneecap dislocate on more than one occasion?
(5)	Do you consider yourself double jointed?

Adapted from [117].

**Table 10:** Lifestyle recommendations for JHS/EDS-HT.

Recommendation	
Promote regular, aerobic fitness	
Promote fitness support with strengthening, gentle stretching, and proprioception exercises	
Promote postural and ergonomic hygiene especially during sleep, at school, and at workplace	
Promote weight control (BMI < 25)	
Promote daily relaxation activities	
Promote lubrication during sexual intercourse (women)	
Promote early treatment of malocclusion	
Avoid high impact sports/activities	
Avoid low environmental temperatures	
Avoid prolonged sitting positions and prolonged recumbency	
Avoid sudden head-up postural change	
Avoid excessive weight lifting/carrying	
Avoid large meals (especially of refined carbohydrates)	
Avoid hard foods intake and excessive jaw movements (ice, gums, etc.)	
Avoid bladder irritant foods (e.g., coffee and citrus products)	
Avoid nicotine and alcohol intake	

Adapted from [118].

Note: these recommendations must be intended as flexible indications for ameliorating quality of life and do not represent lifesaving solutions.
